# Etiological Analysis of Viral Encephalitis in Children in Zhejiang Province from 2018 to 2019

**DOI:** 10.3390/diagnostics12081964

**Published:** 2022-08-14

**Authors:** Juan-Juan Liu, Li-Ping Teng, Chun-Zhen Hua, Yong-Ping Xie, Yan-Xiang Pan, Bo-Fei Hu, Wei-Lin Hu, Wei-Jian Wang

**Affiliations:** 1 Department of Infectious Diseases, National Clinical Research Center for Child Health, Children’s Hospital, Zhejiang University School of Medicine, Hangzhou 310052, China; 2 Department of Rheumatology Immunology and Allergy, National Clinical Research Center for Child Health, Children’s Hospital, Zhejiang University School of Medicine, Hangzhou 310052, China; 3 Department of Clinical Laboratory Center, National Clinical Research Center for Child Health, Children’s Hospital, Zhejiang University School of Medicine, Hangzhou 310052, China; 4 Department of Medical Microbiology, Zhejiang University School of Medicine, Hangzhou 310058, China; 5 Department of Research and Development, Health Gene Technologies Co., Ltd., Ningbo 315000, China

**Keywords:** viral encephalitis, etiology, children, multiplex RT-PCR

## Abstract

Objective: To investigate the common pathogens of viral encephalitis (VE) in children, and to provide guidance for the empirical diagnosis and treatment of patients with VE. Methods: A total of 227 cerebrospinal fluid (CSF) samples were collected from pediatric patients with VE in Zhejiang province from January 2018 to December 2019. The samples were tested using multiplex and singleplex Reverse Transcription-Polymerase Chain Reaction (RT-PCR) with primers specific to enterovirus (EV), varicella-zoster virus (VZV), mumps virus (MuV), cytomegalovirus (CMV), herpes simplex virus type 1 (HSV-1)/type 2 (HSV-2), Epstein–Barr virus (EBV), and human herpesvirus 6 (HHV-6). The data of the two analyses were compared and then verified using Sanger sequencing. Results: Of the 227 CSF samples, 90 were shown to be positive for multiplex RT-PCR with a positivity rate of 39.65% and a 95% confidence interval (33.2%, 46.1%). EV was the most common cause of VE, followed by EBV, HHV-6, MuV, CMV, VZV, and HSV-1. Most included cases occurred in summer, accounting for 49.78% of all cases. For EV, EBV, and HSV-2, multiplex RT-PCR showed a positivity rate of 34.36%, which was not statistically different from that of 30.4% shown by singleplex RT-PCR. The sequences of EV, EBV, VZV, MuV, CMV, HSV-1, HHV-6, and HSV-2 were confirmed by sequencing the PCR products obtained from multiplex and singleplex PCR. Conclusions: In children, VE is more prevalent in the summer than in other seasons in Zhejiang province, and EV may be the most common causative pathogen.

## 1. Introduction

Viral encephalitis (VE), a common infectious disease of the central nervous system (CNS) in children, shows an annual incidence of approximately 10.5/100,000 worldwide [1]. At least 100 types of viruses are known to cause encephalitis [2]. The prognosis of VE differs depending on the causative organism [3,4]. For example, the VE caused by the mumps virus (MuV) is often mild with a good prognosis, does not require antiviral therapy, and resolves on its own [4]. However, the VE caused by herpes simplex viruses (HSVs) is often severe, as the condition progresses rapidly and requires antiviral treatment such as acyclovir with high mortality. Survivors of VE induced by HSVs often have varying degrees of neurological sequelae [3]. The clinical manifestations of VE vary in severity, so treatment strategies should differ depending on the causative pathogen. Therefore, early identification of the causative pathogen is important in the treatment, management, and prevention of VE.

The routine viral nucleic acid assays, currently used by most hospitals in China, are limited to the detection of several viruses such as enterovirus (EV), Epstein–Barr virus (EBV), and HSV type 2 (HSV-2). Additionally, these Polymerase Chain Reaction (PCR) assays can only screen for one virus at a time with multiple steps, straining the personnel and laboratory resources already limited by the coronavirus disease 2019 (COVID-19) pandemic. Therefore, herein we employed multiplex Reverse Transcription-PCR (RT-PCR) to simultaneously detect the nucleic acids of eight viruses in the cerebrospinal fluid (CSF) samples collected from pediatric patients with VE. The results obtained in our present study will simplify and expand the scope of multi-pathogen detection and provide a reference for the empirical diagnosis of patients with VE.

## 2. Patients and Methods

### 2.1. Patients

This study was approved by the ethics committee of the Children’s Hospital of Zhejiang University (ID: 2020-IRB-015) and was conducted in accordance with the Declaration of Helsinki. All included patients underwent lumbar puncture (LP) with informed consent signed by their legal guardians. Children aged 0–16 years with VE admitted to the Children’s Hospital of Zhejiang University from January 2018 to December 2019 were enrolled in this research. Inclusion criteria [5]: The prerequisite condition was unexplained mental status changes lasting more than 24 h. Meanwhile, at least one of the following criteria was met: fever ≥38 °C within 72 h of onset; convulsions; manifestations of focal neurologic deficits; alterations in the brains of patients as shown by neuroimaging; electroencephalogram (EEG) abnormality; and CSF clear in appearance with normal or moderate white blood cell count (≥5/mm^3^). Bacterial meningitis was excluded by microscopy and CSF culture. Exclusion criteria were as follows: patient with incomplete medical records; insufficient CSF retention (<1.5 mL); pediatric VE patients diagnosed with immunodeficiencies, autoimmune disease, inherited metabolic disease, multisystem dysfunction, or history of other neurological diseases (including cerebrovascular disease or brain tumors). A total of 227 children were ultimately included in this study. CSF samples were obtained from each pediatric patient within 48 h of hospitalization (basically 2–5 days after onset) using LP with one sample per patient, and the specimens were stored at −80 °C.

### 2.2. RT-PCR

#### 2.2.1. Multiplex RT-PCR

Nucleic acid was extracted from 300 µL of CSF using a Nucleic Acid Extraction Kit (HGT, Ningbo, China) as per the manufacturer’s instructions. The extracted nucleic acid was purified using a Smart LabAssist-16/32 workstation (Taiwan Dot Nanotechnology Co., Ltd., Taichung, Taiwan) per manufacturer’s instructions and immediately used as a template for PCR amplification. The primers for RT-PCR amplification of the eight viruses are shown in Table 1. The workflow of our multiplex RT-PCR system and conditions used in the PCR reaction cycle is described in our previous study [6]. PCR products were then analyzed using advanced fragment analysis (AFA). Briefly, PCR products were transferred into an ABI 3500Dx gene analyzer for capillary electrophoresis. Each PCR product was separated using different migration rates, and the fluorescence intensity of each PCR product was measured. Data were interpreted for positive and negative results using Gene Mapper 4.1 software (Thermo Fisher Scientific, Waltham, MA, USA). LIZ500 was utilized as internal standard during detection by RT-PCR and capillary electrophoresis.

#### 2.2.2. Clinical Singleplex PCR for the Detection of Viral Nucleic Acids

RT-PCR was used for EV RNA detection, performed by Shanghai ZJ Bio-Tech Co., Ltd. PCR for detection of EBV and HSV-2 was performed by Daan Gene Co., Ltd. Reactions were performed in an ABI StepOnePlus PCR system per manufacturer’s instructions. For the detection of EV, conditions for RT-PCR reactions were as follows [7]: 50 °C for 15 min; 95 °C for 5 min; 40 cycles at 94 °C for 15 s and at 55 °C for 45 s. CT values < 35.0 were considered positive. For the detection of EBV, conditions for PCR reactions were as follows [8]: 93 °C for 2 min; 40 cycles at 93 °C for 15 s and at 55 °C for 45 s. CT values < 37.0 were considered positive. For the detection of HSV-2, the PCR conditions were 93 °C for 2 min; 40 cycles at 93 °C for 15 s and at 55 °C for 45 s. CT values < 37.0 were considered positive.

### 2.3. Verification of Results Obtained Using Multiplex RT-PCR and Singleplex PCR

Next, the PCR products were sequenced to confirm the presence of EV, EBV, VZV, MuV, CMV, HSV-1, HHV-6, and HSV-2 in our samples of CSF. The PCR products subjected to verification included: 7 multiplex RT-PCR products for EV, EBV, VZV, MuV, CMV, HSV-1, and HHV-6 with 1 PCR product per each virus; 3 singleplex PCR products for EB, EV, and HSV-2. The genes targeted for verification included UL52 of HSV-1, 5′ non-coding region (5′UTR) of EV [9], genome structure repeat region 3–9 (repeat region 3–9) of EBV, UL54 of CMV, UL52 of HSV-2, U4 of HHV-6, ORF21 of VCV, and V/P of MuV. Table 2 shows the primers used for amplification of the target genes described above. The 10 PCR-amplified products described above were sequenced by Tsingke Biotechnology (Hangzhou, Zhejiang, China) and then analyzed using NCBI BLAST.

### 2.4. Statistical Analysis

Data were analyzed using SPSS 18.0 statistical software. Chi-square test was used for the analysis of gender ratio of the patient population and seasonal distribution of EV and Paired chi-square test for comparison of the results of multiplex RT-PCR and singleplex PCR. Receiver operating characteristic (ROC) curve and the area under the ROC curve (AUC) were used to assess the diagnostic power of multiplex RT-PCR. One-way analysis of variance (ANOVA) was used to compare the average age of onset among the three groups of viruses (EV, EBV, and HHV-6). Pairwise comparison between groups was performed using the Student–Newman–Keuls (SNK) method. *p* < 0.05 was considered statistically significant.

## 3. Results

### 3.1. Multiplex RT-PCR Virus Detection

Of the 227 CSF samples from 227 pediatric patients manifesting clinical symptoms of VE, 90 samples tested positive with a positivity rate of 39.65% (90/227) and a 95% confidence interval of (33.2%, 46.1%). EV had the highest positivity rate of 32.16% (73/227), followed by that for EBV (3.96%, 9/227), HHV-6 (2.64%, 6/227), MuV (0.88%, 2/227), CMV (0.88%, 2/227), VZV (0.44%, 1/227), and HSV-1 (0.44%, 1/227), while HSV-2 was not detected. Four of the 90 etiologically positive cases were co-infected with EV and EBV.

#### 3.1.1. Comparison of Virus-Detection Rate in Patients of Different Gender and Age

**Figure 1:**
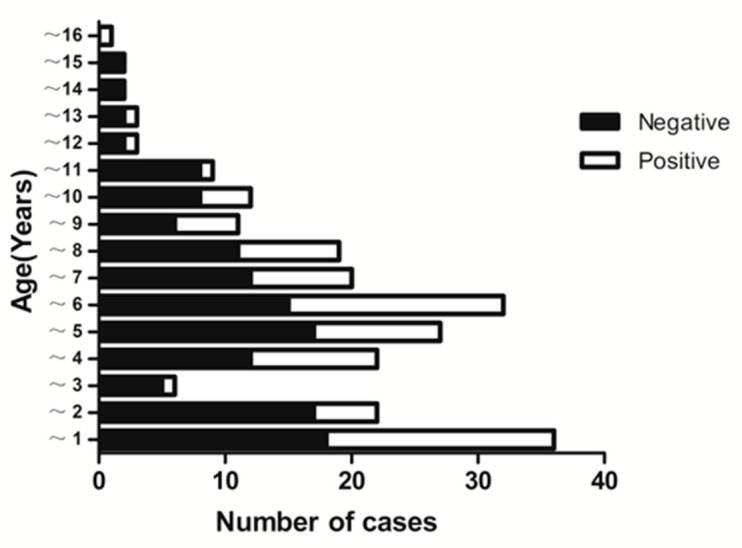
Distribution of childhood viral encephalitis by age and virus-detection results.

In this study, the ratio of boys to girls was 1.46:1. The age of the patients was normally distributed, with a mean age of 60.09 months, minimum age of 7 days, and maximum age of 15 years and 7 months. The rate of pathogen detection in the CSF samples showed no significant differences in gender (X^2^ = 0.177, *p* > 0.05). The number of VE cases under 1 year old was the largest, occupying 15.86% of all participants (36/227). Of these 36 infants, 18 were positive for etiology, consisting of 14 cases of EV (all less than 3 months) and 4 of HHV-6. Figure 1 depicted no statistical difference in the positivity rate of pathogen detection among age groups. For the three viruses showing the highest positive-detection rates (EV, EBV, and HHV-6), Table 3 indicated significant differences in the average age of onset among groups (*p* < 0.05).

#### 3.1.2. Comparison of Virus Detection Rate with Respect to Seasonality

**Figure 2:**
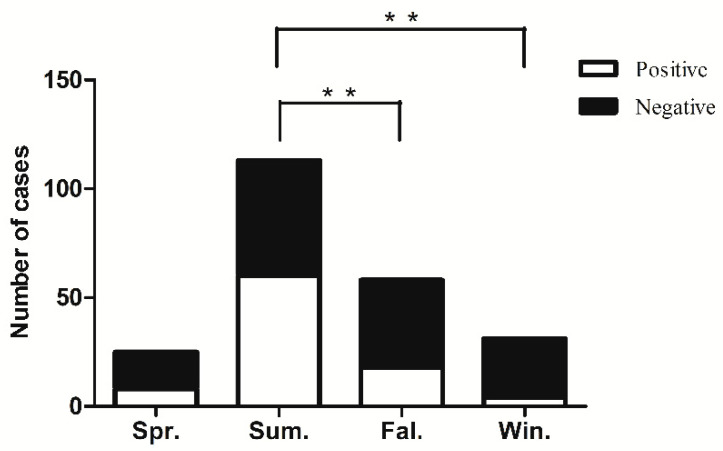
Seasonal distribution of viral encephalitis cases in children. Positive virus detection rate varied significantly with respect to different seasons. ** *p* < 0.008.

The incidence of VE was highest in the summer, accounting for 49.78% (113/227) of the cases in Zhejiang province. For this high summer prevalence, EV was the largest contributor, responsible for 47.79% (54/113). As shown in Figure 2, the rate of positive virus detection varied significantly with respect to different seasons (X^2^ = 20.22, *p* < 0.01). Pairwise comparison showed that the pathogen detection rate was significantly higher in summer than autumn and winter (*p* < 0.008).

### 3.2. Comparison of Viral Detection Rates Using Multiplex and Singleplex RT-PCR

**Figure 3:**
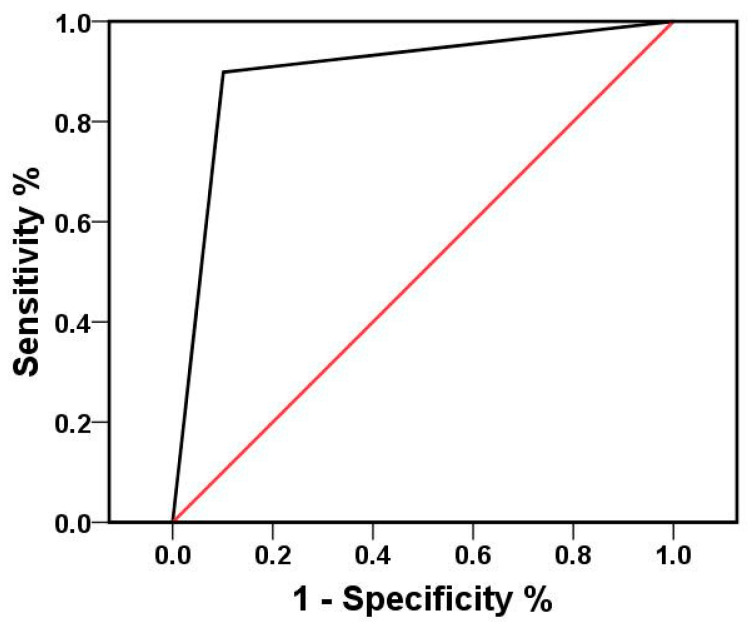
ROC curve: ROC of multiplex RT-PCR. Diagnostic value of multiplex RT-PCR. Taking singleplex RT-PCR results as the gold standard, the AUC of multiplex RT-PCR was 0.9, which was statistically significant compared with 0.5 (*p* < 0.01). ROC, Receiver operating characteristic; AUC, area under the ROC curve.

Analysis of the 227 CSF samples by the routine clinical singleplex PCR detected only the presence of EV, EBV, and HSV-2, while of the 69 positive samples, 67 were positive for EV, 1 for EBV, and 1 for HSV-2. The positive detection rate for these pathogens was 30.4% (69/227), while the rate for EV, EBV, and HSV-2 demonstrated by multiplex RT-PCR was 34.36% (78/227). Thus, the difference in the detection rate of EV, EBV, and HSV-2 between multiplex and singleplex RT-PCR was not statistically significant (*p* = 0.093, McNemar test). In addition, ROC curve showed that the diagnostic accuracy of multiplex RT-PCR was high (AUC, 0.9; 95% confidence interval, 0.85 to 0.95) (Figure 3).

### 3.3. Verification of RT-PCR Products Using Sanger Sequencing

The 10 obtained PCR products were verified using Sanger sequencing. The sequencing results were then compared using BLAST in the NCBI database. The PCR products showed a similarity of 98.89–100% with the sequences available in the NCBI database.

## 4. Discussion

Early identification of the cause of VE is essential for determining treatment strategies and reducing unnecessary examinations, and empirical treatment. A rapid, sensitive, and comprehensive test for the common infectious encephalitis pathogens requiring only a small amount of CSF would be very useful [10]. In this study, multiplex and traditional singleplex PCR were used to detect pathogens in CSF. The reliability of the results obtained using multiplex and singleplex PCR was confirmed by Sanger sequencing. There was no significant difference in the detection rate between multiplex RT-PCR and traditional singleplex PCR. However, multiplex RT-PCR could simultaneously detect eight viruses in a reaction tube, which eliminates the requirement for additional CSF and saves time. Compared with traditional singleplex PCR, multiplex PCR not only reduces the workload of experiment, but also avoids the possibility of contamination of samples due to repeated experiments. Meantime, the detection results of multiple pathogens can be fed back to the clinician at one time, reducing the time cost of communication between the laboratory and the clinician and making pathogens’ diagnosis faster. Currently, the traditional singleplex PCR detection in our hospital has only carried out the detection of HSV-2, EV and EBV, which cannot meet upsurging clinical needs. The multiplex RT-PCR detection in the study could detect eight viruses, including HSV-1, HHV-6, and MuV, etc., which remedies the defect of the narrow pathogen spectrum of singleplex RT-PCR in our hospital. Our results show that multiplex RT-PCR allowed for rapid identification of multiple pathogens and, therefore, has broad application potential.

In this study, EV was indicated as the most common cause of VE in patients admitted to our hospital, followed by EBV and HHV-6 (the second and third most common VE-causing viruses, respectively). Of course, the pathogen spectrum shows differences with respect to region and population. A study from the US showed that HSV and EV were the main causes of VE [3], and another study from hospitals in northern China reported that EV, MuV, and HSV-1 were the top three most common causes of VE [11]. The differences between our findings and the previous works may be related to yearly and seasonal differences in viral distribution patterns, and the region in which each separate study was conducted.

The VE examined in our study showed a seasonal increase in summer, which is consistent with the peak time of hand-foot-mouth disease, and is likely related to the increased temperature and humidity of the summer season [12,13]. Additionally, our results indicate that EV was the primary pathogen of VE examined. Infection with EV produces high-titer viremia, which spreads from the primary part of the intestine or respiratory tract to the CNS, leading to various pathological manifestations [14]. A total of 3% of neurological cases presented with severe encephalitis [15]. Multiplex PCR could be useful for detecting the condition, contributing to speedy diagnosis and timely targeted treatment.

VE is most common in children under 1 year of age [16]. Our study also suggested the highest number of VE cases was under 1 year old (36/227). The positivity rate of EV was the highest among the 36 infants, with 14 cases of EV encephalitis less than 3 months. The prevalence of EV-induced encephalitis in children younger than 3 months is 7.7% [17]. The above results indicated that EV-induced encephalitis was particularly common in young children. This phenomenon suggests a causal relationship between the development of the neuroimmune system and the neuro invasion of EV. In addition, parechovirus caused encephalitis can develop the septic-like disease in neonates and infants [17]. It is difficult to distinguish parechovirus encephalitis from other infectious diseases in this age group based only on patient’s clinical symptoms. In other words, if children below 3 months of age show clinical manifestations of fever, lethargy, reduced appetite, crying, and impaired movement, admission to a medical facility for a complete CSF examination and treatment with empiric antibiotics are considered necessary [18]. Early clarification of VE etiology in this population using multiplex RT-PCR is also recommended to shorten the duration of medication use, reduce antibiotic usage, and design a precise treatment strategy.

Neurological damage caused by EBV infection is often underestimated clinically [19]. In this study, EBV accounted for 10% (9/90) of etiologically identified encephalitis cases. Other domestic studies have also shown that EBV is responsible for 5.8–7.98% of etiologically confirmed VE [11,20]. EBV-caused encephalitis (EBE) has no specific clinical manifestations. Therefore, detection of CSF pathogens is necessary. According to this study, EBE mainly occurred in preschool children. However, the pathogenesis of EBE in children is not fully understood. In addition to causing VE, EBV is also associated with severe neurological complications, such as acute cerebellitis, transverse myelitis, and spinal radiculitis, which show an incidence of 5.2% [21,22]. Thus, detection of EBV in the CSF has some clinical significance.

HHV-6-caused encephalitis occurs mostly in infants and young children. HHV-6 is a lymphotropic and neurotrophic virus subcategorized into HHV-6A and HHV-6B [23]. In children with a healthy immune function, primary infection with HHV-6B is associated with various clinical forms of encephalitis [24]. A recent meta-analysis showed that infection with HHV-6 accounts for 21% of children presenting with fever and convulsions, with an average age at disease onset of 16.24 months [25]. Another study reported 23 cases of HHV-6 among the 405 CSF samples obtained from VE patients less than 2 years old in Zhejiang, with a detection rate of 5.68% [26]. In our present study, we detected 6 cases of HHV-6 among the 227 CSF specimens, and 4 of these patients were ≤1 year old. These findings suggest that clinicians should be aware of possible HHV-6 infections in infants and young children presenting with fever and convulsions. The multiplex RT-PCR technique utilized in our present study might quickly identify HHV-6 and other pathogens causing VE in our pediatric patients.

In addition to EBV and HHV-6, we also detected other herpes viruses in the samples of CSF, such as CMV, VZV, and HSV-1. Among these viruses, HSV-1 is particularly important, because HSV-1 caused encephalitis (HSVE) shows a high mortality rate [27]. Another study has shown that delayed administration of acyclovir in children with HSVE is associated with long-term neurological dysfunction in these patients. Thus, it is advisable that all children with encephalitis undergo a PCR screen for the presence of HSV in the CSF for early diagnosis and treatment [3].

Inevitably, this study had several limitations. As a single-center trial, it could only reflect the etiological characteristics of the VE treated at our facility. Future multi-center studies will increase the reliability of the data utilized in the clinical diagnosis of VE. In addition, numerous other pathogens capable of causing VE in children were not tested in the present study.

In conclusion, VE in children is more prevalent in the summer than in other seasons, and EV may be the most common causative pathogen. Considering the limitations of this study, further development of a convenient and reliable method for multiple pathogen detection is deserved.

## Figures and Tables

**Table 1:** Primer sequences and product sizes of the eight viral genes amplified by multiplex RT-PCR.

Target	Gene	Amplicon Size (bp)	Sequence (5′–3′)
EV	5′UTR	113	F: TATCCACGGCAGAAGTATGAC R: CTGAACTACACTGGGGTTGCT
MuV	V/P	171	F: TTCAGGGAACCAACTCGTTGA R: CTTCGGAGGATGAGACCATGAT
HSV-1	UL52	198	F: CTCTTCGATCGCCCTCCTCAC R: GTCCCCAATAAACAAAAGGT
HSV-2	UL52	207	F: CTCTTCGATCGCCCTCCTCAC R: GTCCCCAATAAACAAAAGGT
VZV	ORF21	161	F: CAACTCTCTATTATGATGAAC R: AAGATGTGGCGAAGGATAAA
EBV	Repeat region 3–9	228	F: CTGACACTTTAGAGCTCTGGAG R: GGCCCTGACCTTTGGTGAAGTCA
HCMV	UL54	182	F: CCGGAGCCGACGCTGAAAACGCC R: GCCGTGTCACCTAACGCCGCTAT
HHV-6	U4	274	F: TTGGGCAAAATATCGAACAGGGT R: AGACGACCATGGATCTCTCGGC

**Table 2:** Target genes and primers used for amplification and verification of the eight viral products using Sanger sequencing.

Target	Gene	Amplicon Size (bp)	Sequence (5′–3′)
HSV-1	UL52	432	F: CTTTATGCGACCGACGGGTG R: GATGGCCACGGTGAGAGC
EV	5′UTR	122	F: AGTCCTCCGGCCCCTGAATG R: GAAACACGGACACCCAAAGTA
EBV	Repeat region 3–9	470	F: ACCACTTTATACCAGGGGCAG R: AAGTAGAGGCTCAGGCCATG
HCMV	UL54	371	F: GTATTGGTGCGCGATCTGTTCA R: CCGACGCTGCTACTACTGTTACT
HSV-2	UL52	459	F: ATTACGTCTACGCTGTTGCT R: GGCGGAAAAGTTGAAGTTSAGG
HHV-6	U4	554	F: GTATCCCTCTTCTCCCCATCGAC R: ATGAGTGATTGTGTGYTTTCTCG
MuV	V/P	413	F: CAACYATCCAACAACAGGTTCATA R: ATTTGGGTCACCATGCTGCC
VZV	ORF21	344	F: CTTTGGTTCCTGACCGTCCTT R: ATTCCCACTCTCATTTGCGTTC

**Table 3:** Age distribution in children with positive virus-detection results.

Group	Number of Cases N = 90	Age (Years) Mean ± SE	F	*p*
EV	69	4.64 ± 0.37		
EBV	5	7.22 ± 1.04	3.97	0.023 *
HHV-6	6	2.06 ± 1.20		0.006 **
HCMV	2	3.57 ± 3.50		
MuV	2	7.04 ± 1.88		
HSV-1	1	1.17		
VZV	1	15.58		
EV + EBV	4	4.72 ± 0.29		

* Comparison of onset ages among EV, EBV, and HHV-6 groups. ** The pairwise comparison among EV, EBV and HHV-6 groups suggested a statistical difference in the onset age of EBV and HHV-6 (*p <* 0.017), while there was no statistical difference in the onset age of other groups.

## Data Availability

Data supporting the findings of this study are available from the corresponding author. The data cannot be made public because of privacy or ethical restrictions.
